# Remarks on the Importance of Avoiding the Contamination of Water with Lead

**Published:** 1858-07

**Authors:** James Bower Harrison

**Affiliations:** Corresponding Member of the Epidemiological Society of London.


### REMARKS ON THE IMPORTANCE OF AVOIDING THE CONTAMINATION OF WATER WITH LEAD.

By JAMES BOWER HARRISON, M.D., F.R.C.S., Corresponding Member of the Epidemiological Society of London.

It is now many years ago since I first directed my attention to the subject of the contamination of water with lead. Some cases which occurred in my practice strongly impressed me with its importance. I could not but remark with interest the insidious nature of a poison which is ever present in our dwellings; the stealthy advances of the disease which it occasions; the fearful consequences which ensue; and above all, the happy manner in which the patient may be snatched from an impending and miserable death.

A subject of great antiquity and singular importance seemed to have fallen into comparative neglect; numerous writers had recorded with diligence, accuracy and even prolixity, the evil consequences which arose to artisans in the various occupations in which lead was employed. The maladies of plumbers and painters had been like household words. All the while the effects of lead on water were at once known and forgotten, the subject imperfectly understood and practically neglected. In 1803, Dr. Lambe, of Warwick, wrote a work entitled *Researches into the properties of Spring Water, with medical cautions against the use of Lead.* It was dedicated to Sir George Baker, and intended as a supplement to the able inquiries of that distinguished physician into the Devonshire colic. We must not measure the value of Dr. Lambe's work by the scientific accuracy of his conclusions. The merit of research does not rest with the last step of the inquiry. Dr. Lambe entered on the investigation rather as a medical philosopher than as a chemist; but because he *did* enter on the inquiry, he has done good service to the profession and to mankind. The inquiry may be said to have slept again after the treatise of Dr. Lambe. Perhaps his errors may, in some degree, have retarded the progress of truth. From time to time, individual inquirers recorded the deleterious effects of water impregnated with lead, but all the while lead was constantly being used, and its effects as constantly being produced. It is only now that we are awakened to the full importance of the subject. I have myself endeavoured to shed some light upon the inquiry, to press it upon the attention of others, and to collect together the scattered fragments of information which are to be found. Again and again I have been asked to witness, in my own practice, the effects of the lead-poison, and trace their slow and painful progress. Mr. Roberton, of Manchester, has lately directed my attention to the results of his own experience, and urged me to continue an investigation which has so warmly interested me. I shall not go over again the details which I have already published,* I now only seek to collect into a focus the most interesting particulars which are elicited. It is the domestic evils which I have chiefly sought out, and it is to the domestic evils to which I would again refer. When first I resided in Broughton, the supply of water there was exceedingly defective; many families made use of soft water, collected in leaden cisterns, for every purpose for which water was required. Illness was a natural result, *lead colic* was frequent, and many persons left the neighbourhood with the full conviction that the air was insalubrious. In vain I pointed out the deleterious nature of the contaminated water; often my labours were ridiculed and my injunctions disobeyed. My striking success in the event of many cases sustained my interest on the subject. I looked always to discover the poison of lead when the symptoms pointed in that direction. I had considered these symptoms with careful deliberation, and found them identical with those which arise on the artificers of lead, and which have been so faithfully described by Desplanches. I noticed the sallow complexion of the skin, the constipation and griping of the bowels, the paralysis of the limbs, and the various other indications. Wherever these existed, I looked everywhere for lead, and abandoned the search only when every possible inquiry had been made. A thorough knowledge of the symptoms of lead disease seemed to me the foundation and very starting point of each investigation. If the symptoms existed, it was not sufficient to examine the water which might be supplied for analysis, or which the patient might desire his domestics to produce. It was necessary to find *the water* which had occasioned the complaint. I did not seek it, therefore, from the hands of the domestics and friends of patients. I took it from tea-kettles and water-bottles. In this way I prevented both error and deception, and arrived at once at a solution of the mystery. I have said that a knowledge of the symptoms of lead disease was the starting point of each inquiry. I may add that these symptoms—the true exponents of the evil—have not been properly understood. The language of disease has not been fairly interpreted, and its lineaments have remained unrecognized. It is thus that cases have been passed over which might easily have been understood and as easily remedied. It may *yet* be that the extent of the evil is unknown, that future inquirers may still have to discover its hidden ramifications.

* On the Contamination of Water with Lead. London: Churchill. 1852.

